# Intra-tumor heterogeneity in TP53 null High Grade Serous Ovarian Carcinoma progression

**DOI:** 10.1186/s12885-015-1952-z

**Published:** 2015-11-30

**Authors:** Alba Mota, Juan Carlos Triviño, Alejandro Rojo-Sebastian, Ángel Martínez-Ramírez, Luis Chiva, Antonio González-Martín, Juan F. Garcia, Pablo Garcia-Sanz, Gema Moreno-Bueno

**Affiliations:** 1 Departamento de Bioquímica, Universidad Autónoma de Madrid (UAM), Instituto de Investigaciones Biomédicas “Alberto Sols” (CSIC-UAM), IdiPAZ, Madrid, Spain; 2 MD Anderson International Foundation, Madrid, Spain; 3 Sistemas Genómicos, Valencia, Spain; 4 Department of Pathology, MD Anderson Cancer Center, Madrid, Spain; 5 Department of Molecular Cytogenetics, MD Anderson Cancer Center, Madrid, Spain; 6 Department of Gynecologic Oncology, MD Anderson Cancer Center, Madrid, Spain; 7 Department of Medical Oncology, MD Anderson Cancer Center, Madrid, Spain

**Keywords:** Ovary cancer, High grade serous carcinoma, *TP53* null, Intra-tumor heterogeneity

## Abstract

**Background:**

High grade serous ovarian cancer is characterised by high initial response to chemotherapy but poor outcome in the long term due to acquired resistance. One of the main genetic features of this disease is *TP53* mutation. The majority of *TP53* mutated tumors harbor missense mutations in this gene, correlated with p53 accumulation. *TP53* null tumors constitute a specific subgroup characterised by nonsense, frameshift or splice-site mutations associated to complete absence of p53 expression. Different studies show that this kind of tumors may have a worse prognosis than other *TP53* mutated HGSC.

**Methods:**

In this study, we sought to characterise the intra-tumor heterogeneity of a *TP53* null HGSC consisting of six primary tumor samples, two intra-pelvic and four extra-pelvic recurrences using exome sequencing and comparative genome hybridisation.

**Results:**

Significant heterogeneity was found among the different tumor samples, both at the mutational and copy number levels. Exome sequencing identified 102 variants, of which only 42 were common to all three samples; whereas 7 of the 18 copy number changes found by CGH analysis were presented in all samples. Sanger validation of 20 variants found by exome sequencing in additional regions of the primary tumor and the recurrence allowed us to establish a sequence of the tumor clonal evolution, identifying those populations that most likely gave rise to recurrences and genes potentially involved in this process, like *GPNMB* and *TFDP1*. Using functional annotation and network analysis, we identified those biological functions most significantly altered in this tumor. Remarkably, unexpected functions such as microtubule-based movement and lipid metabolism emerged as important for tumor development and progression, suggesting its potential interest as therapeutic targets.

**Conclusions:**

Altogether, our results shed light on the clonal evolution of the distinct tumor regions identifying the most aggressive subpopulations and at least some of the genes that may be implicated in its progression and recurrence, and highlights the importance of considering intra-tumor heterogeneity when carrying out genetic and genomic studies, especially when these are aimed to diagnostic procedures or to uncover possible therapeutic strategies.

**Electronic supplementary material:**

The online version of this article (doi:10.1186/s12885-015-1952-z) contains supplementary material, which is available to authorized users.

## Background

Ovarian cancer is the most common cause of death from gynecological malignancies. Around seventy percent of ovarian cancers are histologically classified as high-grade serous carcinoma (HGSC). The standard treatment for these tumors is cytoreductive surgery followed by platinum-taxane chemotherapy. Although the initial response rate is higher than 80 %, the majority of patients relapses within five years and die due to chemo-resistant disease [1, 2].

HGSCs are characterised by nearly universal *TP53* gene mutation, present in more than 95 % of the cases [3]. The most common *TP53* abnormalities are missense mutations, which induce nuclear accumulation of the mutant protein with a strongly positive IHC staining. However, approximately 30 % of somatic *TP53* mutations are considered null mutations that lead to complete absence of p53 protein due to nonsense, frameshift or splicing junction mutations [4]. The prognosis value of *TP53* status is a controversial issue [5]. Nevertheless, several studies support that tumors with *TP53* null mutations present a worse outcome compared with those in which *TP53* harbors mutations involving overexpression. This would happen not only in ovarian cancers [6–9], but also in breast, colorectal and head and neck cancers and leukemia [10].

The Cancer Genome Atlas (TCGA) consortium has enabled a deeper understanding of HGSCs using an integrated genomic approach for the analysis of 316 tumors. This study has revealed that, with the exception of *TP53* mutation, present in 96 % of the tumors, recurrent mutations are not common in HGSCs. Nonetheless the hallmark of HGSCs was numerous somatic copy number alterations, with more than 100 recurrent amplifications and deletions identified [11]. However, this analysis only considered primary tumors samples, regardless of subsequent recurrences. In fact, there are few studies that have analysed from a genomic point of view the evolution of these tumors using paired samples of primary tumor and post-treatment relapse. According to some of these studies, primary and recurrent diseases would be genetically similar [12–14]. Conversely, other studies have shown a high degree of intra-tumor heterogeneity, which would allow clonal evolution and the successive tumor progression and resistance to chemotherapy [15, 16]. In fact, intra-tumor heterogeneity has been shown to be intrinsic to primary tumor and not just a result of chemotherapy treatment [14, 17].

To the best of our knowledge, none of these studies considered *TP53* null tumors as an individual clinical entity. In the present study, whole-exome sequencing and comparative genomic hybridisation was performed on primary tumor and recurrence implants of a *TP53* null patient, revealing significant heterogeneity between all samples. Selected variants detected in the genomic analysis were subsequently validated in multiple tumoral implants from the primary tumor and the recurrence. These data revealed intra-tumor heterogeneity in the primary tumor, which was reflected in the different recurrence metastases, suggesting a model of clonal evolution.

## Methods

### Samples selection

**Fig. 1:**
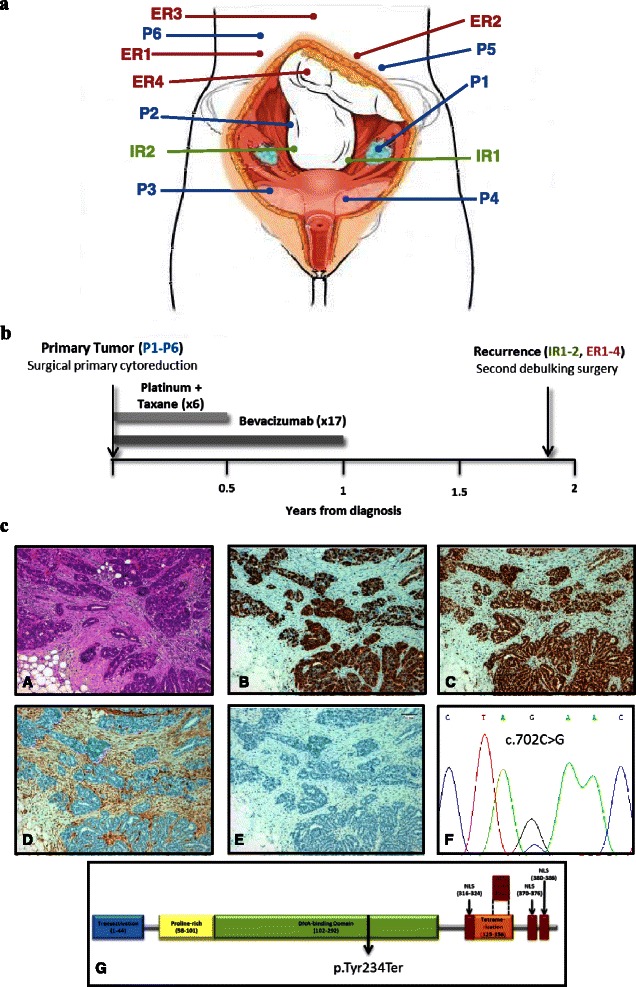
Clinical case and sample description of an ovarian TP53 null HGSC. **a** Anatomical location of the primary tumor (blue) and recurrence samples (intra-pelvic samples in green and extra-pelvic samples in red). **b** Patient’s clinical course. Grey squares indicate periods of treatment between primary tumor diagnosis and relapse. **c** Representative images of hematoxilin/eosin staining (A) and immunohistochemistries for WT1 (B), Ki-67 (C), PTEN (D) and p53 (E). TP53 mutation detected by Sanger sequencing (F) and its consequence in p53 protein (G)

Tumoral samples from primary and recurrence disease were selected from a TP53 null case of HGSC. Biospecimens and clinical data were collected after written approval of the study by the Ethics Committee of the MD Anderson Cancer Center. A specific informed consent was obtained from the patient managed by the MD Anderson Foundation Biobank (record number B.0000745, ISCIII National Biobank Record) and included the authorization for data publication of individual clinical data and any accompanying images. A total of twelve samples were selected, three frozen and nine Formalin-Fixed Paraffin-Embedded (FFPE) tissue. Six primary tumor (P1-P6), two intra-pelvic recurrence (IR1-IR2) and four extra-pelvic recurrence (ER1-ER4) implants were analysed (Fig. 1a). All samples selected contained at least 80 % of tumoral cells. Non-tumoral mesothelium (N) was used as control tissue.

### Immunohistochemistry

Immunostaining was performed on 2 μm FFPE tissue sections. Deparaffinisation and antigen retrieval were done in a PT Link module (Dako, Denmark) and immunohistochemistry was done in an Autostainer (Dako). Primary antibodies used were WT1 (clone 6 F-H2, RTU, 1:1, Dako), PTEN (clone 6H2.1, 1:100, Dako), p53 (clone DO-7, RTU, 1:1, Dako) and Ki-67 (clone MIB-1, RTU, 1:1, Dako). Amplification and visualisation of immune complexes and counterstaining were also performed in the Autostainer with the use of an EnVision FLEX+, Mouse (Dako, CA, USA). Slides were counterstained with hematoxylin and evaluated by two independent pathologists.

### Whole-exome sequencing (WES)

Whole-exome sequencing was performed in four frozen samples (N, P1, IR1 and ER1) using the latest version of SureSelectXT Human All Exon V4 + UTR (71 Mb) enrichment kit and SOLiD high-throughput sequencing platform, according to manufacturer’s protocols. Sequencing reactions were carried out to obtain read pairs (75 + 35 nt, paired-end). Data quality was estimated using parameters from SETS (SOLiD Exerimental Tracking System) software. For the bioinformatics analysis, see Additional file 1: Supplementary Materials.

Variant calling for Single Nucleotide Variants (SNVs) identification was performed using three different algorithms: VarScan2 [18], Mutect [19] and Bioscope. ‘In-house’ scripts were developed to combine the variants and filter possible technical artifacts. SNVs and Indel identified were annotated using the ‘Applicaton Programming Interfaces’ (APIs) from Ensembl v64 and several ‘custom’ scripts. SNVs and Indel with a minimum coverage of 10 and frequency above 0.1 percent were selected. Variants consequences were analysed using Condel [20] (http://bg.upf.edu/fannsdb/), SIFT [21] (http://sift.jcvi.org) and PolyPhen [22] (http://genetics.bwh.harvard.edu/pph2/) predictors.

### SNVs Validation

A total of 25 variants detected by whole-exome sequencing were reanalysed by Sanger sequencing. PCR conditions and length of amplicons are indicated in Additional file 1: Table S2.

### Comparative genomic hybridisation (CGH)

CGH was performed according to Kallioniemi et al. [23], with some modifications. Tumor and normal DNA were labeled using a nick translation kit (Abbott Molecular Inc.). In short, 200 ng of each labeled DNA was hybridised to normal female metaphase cells in the presence of 20–35 mg of Cot-1 DNA for 3 days. After washes, the chromosomes were counterstained with DAPI in an antifade solution. Analysis was performed with a Leica DM4500 epifluorescence microscope equipped with a CCD camera. A minimum of 15 metaphase cells per hybridisation per case were analysed by use of the CytoVision System with version 7.3.1 high-resolution CGH analysis software (Leica Biosystems, UK). The CGH profiles were compared to a dynamic standard reference interval on the basis of an average of normal cases, as previously described [24].

### Fluorescence in situ hybridisation (FISH)

Fluorescence in situ hybridisation was performed as previously described [24]. Briefly, metaphase chromosomes were prepared directly from the same samples used for CGH. Slides were placed at 90 °C for 10 min, dehydrated through a series of ethanol washes, and denatured in the presence of a probe on a plate at 75 °C for 1 min. For detection of gene amplification, gene-specific probes for *PML* and *RARA* (used as control) (Vysis, Downers Grove, IL) were used. At least 100 interphase nuclei were analysed.

### Functional annotation

The selected variants were annotated using David protocols [25, 26]. Briefly, this method allows the functional annotation of genes using different biological database as Biocarta (http://www.biocarta.com/), Gene Ontology (http://geneontology.org/), KEGG (http://www.genome.jp/kegg/pathway.html) and Reactome (http://www.reactome.org/). The interesting functional categories were selected using a p-Value threshold of 0.2. Finally, the correlation between genes and functional categories was represented in form of network using Cytoscape tool [27].

### Hierarchical clustering

A hierarchical clustering method was applied to group the samples on the basis of similarities in mutation pattern. The unsupervised analyses were carried out using the SPSS 17.0 for statistical program (SPSS Inc., Chicago, IL) assuming Euclidean distances between mutations.

## Results

### Clinical history and case description

The patient of this study was diagnosed in 2009 at age 50 with a stage IIIC high grade serous carcinoma (HGSC) with extensive intra- and extra-pelvic peritoneal carcinomatosis. After surgical primary cytoreduction the patient was left with residual disease of less than 1 cm. Afterwards she was included in the OCTAVIA clinical trial [28] and treated with six cycles of weekly paclitaxel and carboplatin, with bevacizumab every 3 weeks for a total period of 12 months. Patient relapsed after 23 months of platinum free interval with numerous tumor implants in intra- and extra-pelvic peritoneum (Fig. 1a). The patient underwent a second debulking surgery without residual macroscopic disease followed by 6 cycles of carboplatin and pegylated liposomal doxorubicin hydrochloride (Fig. 1b).

Both primary and relapsed tumors showed papillary patterns with frequent necrosis, nuclear expression of WT1 and very high proliferative rate (90 % as determined by Ki-67 staining). Immunohistochemistry also showed lack of PTEN expression (suggestive of mutation) and complete absence of p53 staining (indicative of null mutation). Sanger sequencing showed a mutation in exon 7 of *TP53* resulting in a premature stop codon in the position 234 of the protein (Fig. 1c). According to these data, this tumor is classified as a *TP53* null high grade serous carcinoma.

### Identification of somatic nucleotide variants by whole-exome sequencing shows differences in the mutational patterns of distinct tumor regions

**Fig. 2:**
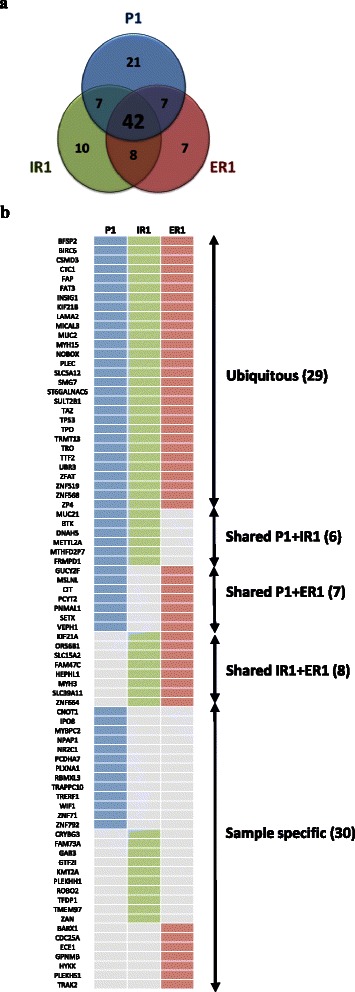
Somatic variants detected by whole-exome sequencing in a TP53 null HGSC. **a** Venn diagram showing the distribution of the total number of variants among the different tumor samples (P1, primary tumor: blue; IR1, intrapelvic recurrence sample: green; ER1, extrapelvic recurrence sample: red). **b** Genes with missense, splice site, nonsense or frameshift mutations with a negative consequence in the corresponding protein and its distribution in the different analysed samples

In order to detect somatic nucleotide variants, whole-exome sequencing was performed in the primary tumor (P1), pararectal recurrence implant (IR1), ileal recurrence implant (ER1) and mesothelium as normal tissue (N) from a *TP53* null patient. After comparison of tumoral samples with the normal tissue, the bioinformatic analysis identified 102 variants, 99 SNVs and three deletions (Additional file 1: Table S1). Only 42 variants were common to all samples, comprising just 41 % of the total. Sample specific variants were identified in the three regions, as well as variants common to two samples (Fig. 2a). Notably, the range of frequencies found in the primary tumor variants suggested the existence of intra-tumor heterogeneity from the onset.

The consequence of each variant in the corresponding protein was analysed by the computational predictors Condel, SIFT and Polyphen [20–22]. A total of 80 variants presented a possibly damaging or deleterious consequence as predicted by at least one of them (Fig. 2b). Interestingly, the scattering of these variants reflects the unequal distribution of the mutations potentially implicated in tumor progression.

A selection of 25 variants detected by WES was reanalysed by Sanger sequencing. Four of them were not validated, showing a false positive rate of 16 % in the WES study (Additional file 1: Table S2). The *TP53* nonsense mutation was detected ubiquitously in the three samples analysed, consistent with the founder role of this gene in this type of cancer.

### Copy Number Variations (CNVs) are unequally distributed among the different tumor regions

**Fig. 3:**
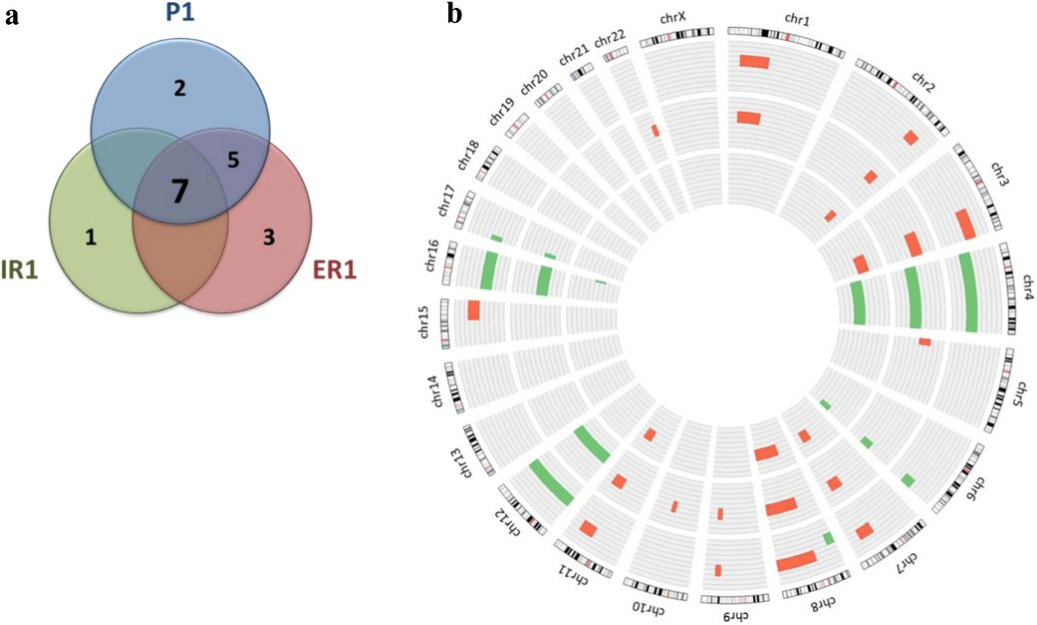
Copy Number Variants (CNVs) detected by comparative genomic hybridisation (CGH) in a TP53 null HGSC. **a** Venn diagram showing the distribution of the total number of copy number changes among the different samples ((P1 primary tumor: blue; IR1,intrapelvic recurrence sample: green; ER1, extrapelvic recurrence sample: red). **b** Circes representation of the copy number changes. P1, ER1 and IR1 samples are displayed in concentric circles (from the outside to the inside). Enhanced regions are represented in red and diminished regions in green

HGSCs are characterised by high chromosomal instability and widespread copy number changes. In order to analyse copy number variations (CNVs), Comparative Genomic Hybridisation (CGH) was performed in the primary tumor (P1) and two recurrence implants (IR1 and ER1), using normal tissue (N) as a reference. We identified changes in 18 chromosomal regions, with gain of material more frequent than loss (61 % vs. 39 %) (Fig. 3, Additional file 1: Table S3). A total of 7 changes (38 % of total) were common to the three samples, counting five gains (2q32q33, 3q22q29, 7q22q32, 8q12q24 and 11q14q22) and two diminished regions (6q25q27 and depletion of whole chromosome 4). P1 and ER1 shared 5 regions (gain of 1p22p35 and 9q31, and loss of 17p13 and depletion of whole chromosomes 12 and 16), while specific changes of each sample were detected (8p22p23 and 15q22q26 in P1; 16q24 in IR1; and 5p15, 10q22 and 22q13 in ER1). Interestingly, no changes exclusively common to P1 and IR1 or to IR1 and ER1 were found.

### The analysis of additional tumor regions shows the existence of mutationally heterogeneous tumor clones

**Fig. 4:**
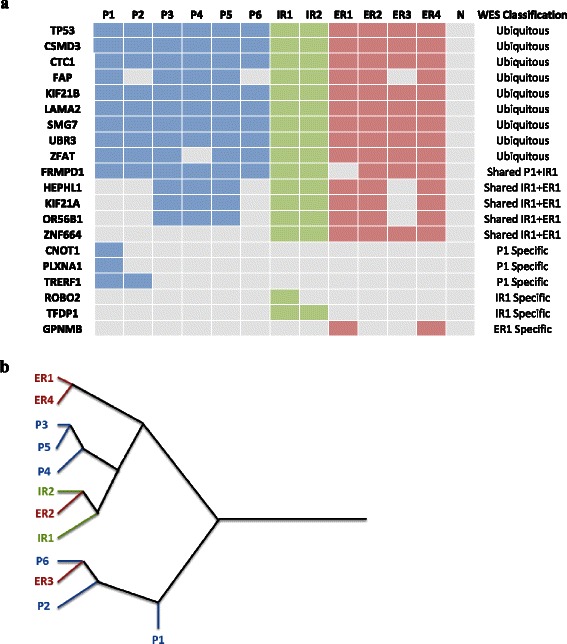
Intra-tumoral mutational pattern and hierarchical clustering to establish a clonal evolution in a TP53 null HGSC. **a** Selected variants detected by whole-exome sequencing were reanalysed in 12 tumoral samples (P: Primary tumor, IR: intrapelvic recurrence, ER: extrapelvic recurrence) and normal tissue (N) by Sanger sequencing. **b** Hierarchical clustering of the analysed samples on the basis of the similarities in mutation pattern

To further investigate intra-tumor heterogeneity, a subset of 20 of the variants detected by exome sequencing was validated by Sanger sequencing in the four original samples (N, P1, IR1, ER1) and in nine additional FFPE samples of tumor implants representing different regions of the primary tumor and recurrences (P2-P6, IR2, ER2-ER4) (Fig. 4a). For the location of the different tumor regions, see Fig. 1a. Variants analysed were selected to represent the different distributions identified in whole-exome sequencing: nine ubiquitous (*TP53*, *CSMD3*, *CTC1*, *FAP*, *KIF21B*, *LAMA2*, *SMG7*, *UBR3* and *ZFAT*), one shared by P1 and IR1 (*FRMPD1*), four shared by IR1 and ER1 (*HEPHL1*, *KIF21A*, *OR56B1* and *ZNF664*), three P1 specific (*CNOT1*, *PLXNA1* and *TRERF1*), two IR1 specific (*ROBO2* and *TFDP1*) and one ER1 specific (*GPNMB*). All these variants were potentially damaging, nonsense, frameshift or splice site variants (Additional file 1: Table S1).

As expected some variants were detected in all of the analysed samples, consistent with the exome sequencing results in which they were detected ubiquitously in P1, IR1 and ER1 samples (Fig. 4a). However, other variants, also ubiquitous according to exome sequencing, were not present in all the samples. Moreover, the *FRMPD1* variant, originally presented in primary tumor and IR1 samples and not in ER1, was current not only in all primary and intra-pelvic regions but also in the other extra-pelvic recurrence samples analysed.

On the other hand, some of the variants initially detected variants as characteristic of them were detected not only in new intra- and extra-pelvic recurrences, but also in primary tumor regions different from the one originally analysed. Moreover, *ZNF664* mutation, (common in IR1 and ER1 samples) was also found mutated in all of the recurrence samples but not in the primary tumor. Specific sample variants were also detected by Sanger sequencing, both for the primary tumor and for intra- and extra-pelvic recurrences, showing a certain degree of mutational heterogeneity among the different tumor locations.

Additionally, intra-tumor genomic heterogeneity was studied by fluorescence in situ hybridisation (FISH) in *PML* gene (15q22), exclusively altered in P1 sample regarding CGH results (Additional file 2: Figure S1). *PML* gain was observed by FISH in P1 but not in IR1 and ER1 samples, confirming CGH data. Furthermore, heterogeneous CNVs were observed in FFPE samples analysed. While P3, P4, P5 and ER1 samples showed 3 or 4 copies of *PML*; P2, P6, IR2 and ER4 presented between 5 and 8 copies. ER3 sample presented just one copy of *PML* per cell, indicating a deletion of this gene in this specific tumor sample (Additional file 2: Figure S1). Therefore intra-tumor heterogeneity was not only observed in genetic variations but also in genomic changes.

### Hierarchical clustering according to mutation pattern allows determining clonal evolution

Since the mutational heterogeneity observed in this tumor is consistent with a clonal composition, we performed a hierarchical clustering analysis based in the distribution of the subset of 20 variants among the 12 different regions of the primary tumor and the two recurrences (Fig. 4b). This analysis shows the existence of two main branches, as shown in the diagram. Most of the recurrences regions would evolve from a primary tumor ancestor subpopulation that gives rise to the upper branch, originating P3, P4 and P5 regions of the primary tumor, the intra-pelvic recurrences and most of the extra-pelvic. Notably, ER2 seems to be more closely related to the intra-pelvic recurrence than to the other extra-pelvic regions. The lower branch contains P1 (the closest region to normal tissue), P2 and P6, and ER3 which would arise from this latter primary tumor region.

Remarkably, it is also possible to hierarchically cluster the three original tumor regions (P1, IR1 and ER1) according to the WES or CGH results. This tumor phylogenetic tree is equivalent to the one generated with the previous subset of 20 mutations, showing two main branches, one corresponding to the primary tumor (P1 region) and the other subdivided in two sub-branches which contain each of the recurrences samples (data not shown).

### Functional annotation and network analysis identifies biological functions implicated in tumor development and progression

**Fig. 5:**
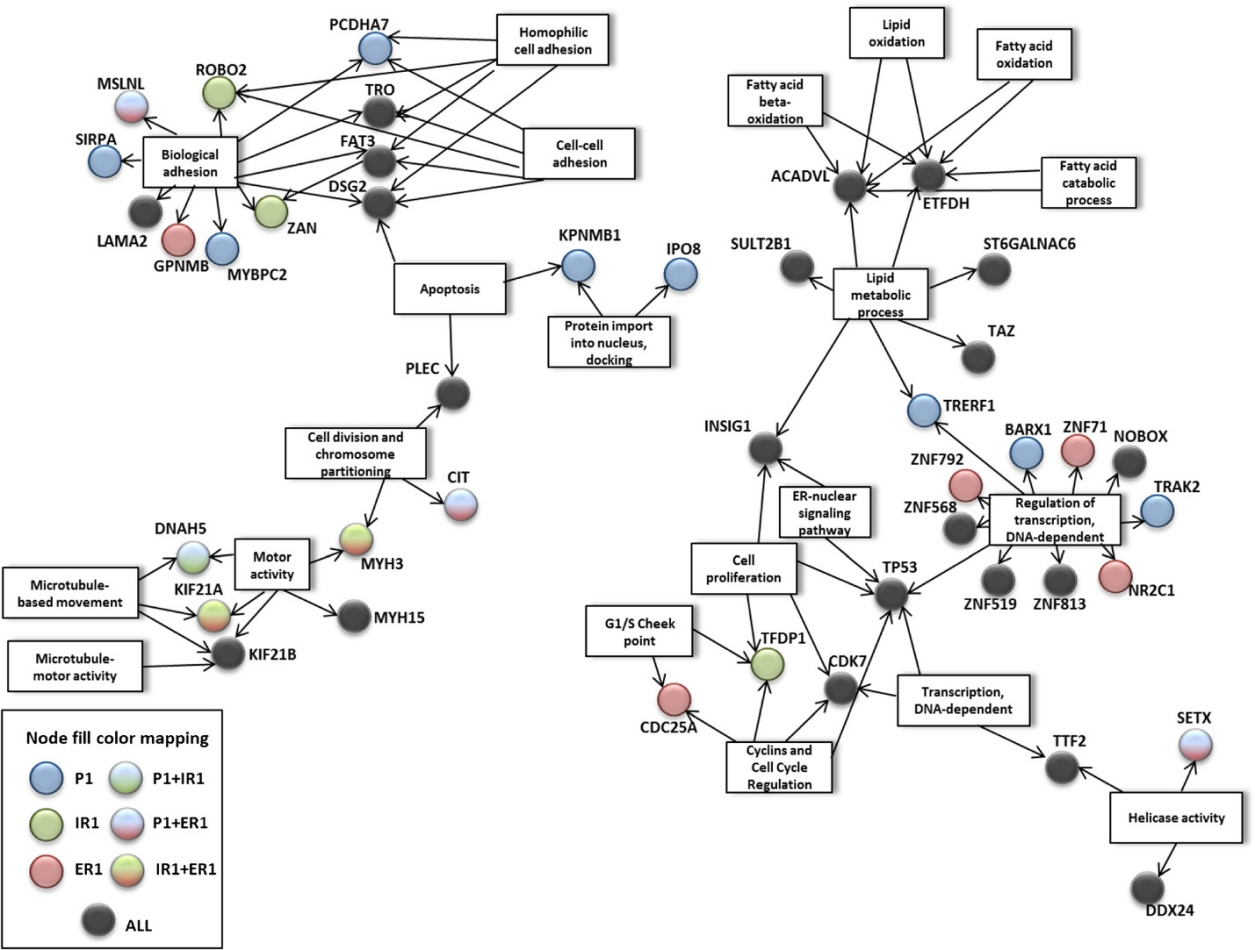
Network analysis of signaling pathways affected by multiple gene mutation. Functional annotation was performed for variants with a possible negative consequence detected by whole-exome sequencing using David protocols (see M&M). Network analysis by Cytoscape tool revealed signaling pathways containing multiple mutated genes. Functional nodes annotated by Biocarta, Gene Ontology, KEGG or Reactome terms are represented in squared nodes. Mutated genes are represented in circles, which colour indicates the samples in which they are mutated (see node fill color mapping)

In order to analyse from a global point of view the biological and molecular functions implicated in the development and progression of this tumor, the functions of the genes mutated according to the exome sequencing were annotated using different biological databases as Biocarta, Gene Ontology, KEGG and Reactome (see Methods section). Network analysis using Cytoscape plugin [27] identified cell adhesion, cell cycle control, microtubule-related movement and transport, lipid metabolism and apoptosis as pathways containing multiple mutated genes (Fig. 5, Additional file 1: Table S4).

Cell adhesion was consistently affected in all the analysed samples, both with mutations common to all samples and with shared or sample specific alterations. Something similar, but with a lower number of affected genes, occurred with cell cycle control, microtubule-based movement, cell division and chromosome partitioning and apoptosis. On the other hand, mutations in genes implicated in lipid metabolism were mainly ubiquitous, pointing to its possible involvement in tumor initiation. In addition, functional annotation was also performed in CGH data, identifying multiple genes implicated in the previous described pathways (data not shown).

Interestingly, although DNA repair pathway is frequently defective in these tumors, we did not identify mutated genes directly related to this pathway. The gene most closely related to this function was SETX, which participates in the defense against oxidative DNA damage [29]. However, CGH data showed that multiple mismatch repair genes, such as *RPA*, *POLE*, *POLD3* or *POLD4*, exhibited copy number changes in all the samples analysed (data not shown).

## Discussion

Despite of initial response to treatment, the majority of HGSC patients will recur and die due to chemoresistant disease. It is well established that a defective status of the DNA repair machinery (especially *BRCA1/2* genes) correlates with a better response to platinum-based therapy [30]. Nevertheless, there is still a huge gap regarding the genetic and molecular factors that contribute to explain the high rate of therapy failure in this disease. Regarding the importance of *TP53* status in this disease, there are evidences pointing to a worse prognosis for tumors with null mutations compared with those in which overexpression inducing mutations are found, although this remains controversial [6–8, 10]. To this respect, a meta-analysis of the genomic data generated by the TCGA consortium regarding ovarian cancer shows decreased overall and progression-free survival for wild type *TP53* tumors with respect to mutant *TP53* cases [31]. Taking this into account, we divided the *TP53* mutant group analysed in the TCGA study into two subgroups: mutant and null tumors. *TP53* null tumors are characterised by complete absence of p53 protein, probably due to degradation of their mRNAs by nonsense-mediated RNA decay [32]. Accordingly, those cases with frameshift, splice site or nonsense mutations and with a mRNA level z-score lower than -1 were classified as *TP53* null tumors. This subgroup shows an intermediate overall survival between the wild-type and mutant groups, with nearly significant and equivalent differences between the different categories (data not shown). In view of that, a deeper understanding of *TP53* null tumors would be needed.

In this study we have analysed by WES and CGH the genetic and genomic alterations of an ovarian *TP53* null HGSC case consisting of the primary tumor, one intra-pelvic and another extra-pelvic recurrence. The exome sequencing analysis shows a significant mutational divergence between the primary tumor and both recurrences. Nevertheless, this degree of divergence decreases when other tumor regions are screened by Sanger sequencing for some of the mutations detected in the exome analysis. The conclusions of this analysis are consistent with a situation in which the primary tumor is composed by mutationally heterogeneous clones, some of which give rise to the recurrences. This heterogeneity is intrinsic to the primary tumor and therefore not expected to be a consequence of the therapy, consistently with what has been previously reported for platinum-based treatment [14]. The hierarchical clustering of the different tumor regions allows to dissect the tumor clonal evolution. The primary tumor regions P3-P5-P4 are the most closely related to the recurrences, with an ancestor tumor subpopulation that would give rise to these primary regions, both intra-pelvic recurrent locations and most of the extra-pelvic (ER1, ER2 and ER4). However, it is worth noting that while the intra-pelvic recurrence seems to have evolved entirely from this ancestor, there is a region of the extra-pelvic recurrence which is more closely related to the other evolutionary branch of the tumor, which gives rise to P1, P2, P6 and, apparently deriving from the latter, ER3. This fact could be regarded as a reflect of a possibly heterogeneous origin for the extra-pelvic recurrence or either as an artifact of the hierarchical clustering process, which does not take into account the CNVs observed in this tumor. Even if the latter is the case, the extra-pelvic recurrence is composed of two distinct subpopulations, one derived from the ancestor clone that gives rise to the upper evolutionary branch (ER1 and ER4) and the other one derived from the intra-pelvic recurrence (ER2), having acquired the ability to migrate to the extra-pelvic area.

As determined by functional annotation and network analysis, cell adhesion and cell cycle control are clearly affected in this tumor. These functions are intrinsic to tumor growth and spread, and therefore its malfunction could be expected. Some of the genes related to cell adhesion altered in this tumor have been previously reported as related to tumor progression. Down regulation and mutations in *ROBO2* have been reported in prostate, gastric and colorectal cancers [33, 34]; while *LAMA2* down regulation has been reported in drug-resistant ovarian cancer cell lines [35]. Moreover the silencing of *TRO*, coding for trophinin, has been related to cisplatin resistance and increased invasiveness of ovarian cancer cells [36]. Conversely *GPNMB*, a migration-related gene, is frequently overexpressed in triple negative breast cancer among others [37]. Interestingly, *GPNMB* is only mutated in ER1 and ER4, suggesting an important role for this gene in the appearance of extra-pelvic recurrence in this patient. Cell proliferation and cell cycle control are also significantly altered in this tumor, not only by gene mutation but also by copy number changes. *TFDP1,* mutated only in the intra-pelvic recurrences regions, participates in cell cycle control modulating E2F pathway, and mutations in this gene can be found in various cancer databases [38]. Moreover, a recurrent frameshift mutation in *TFDP1* has been reported in colorectal cancer [39]. In addition, 8q11.1q24 region, which is amplified in the three samples analysed by CGH, includes among other genes *MYC*, a key inductor of proliferation which has been shown to be commonly amplified in ovarian cancer [11]. Another important pathway consistently affected in all samples analysed was microtubule-based movement. *KIF21A,* one of these mutated genes, has been shown to be down regulated in a murine lung cancer model due to the aberrant methylation of its promoter [40]. It has also been reported, together with other cytoskeleton-associated proteins, to be implicated in breast, cervix and osteosarcoma cells survival [41]. Microtubule-based movement is essential for chromosomal partitioning and segregation, an important role in ovarian HGSC, characterised by wide genomic and chromosomal aberrations. This specific case shows extensive copy number variations, with depletion of whole chromosomes 4, 12 and 16. More unexpected was the alteration of lipid metabolism. Mutations related to lipid metabolism were mainly shared by the three samples analysed by WES, suggesting a driver role for this pathway. Some of the mutated lipid-related genes have been reported to be involved in tumor progression in other types of tumors. *ACADVL* expression is down regulated in adrenocortical tumors [42]. Germline polymorphisms in the estradiol-metabolism related enzyme *SULT2B1* have been associated to prostate cancer risk [43], and its expression has been shown to be reduced in this type of tumor [44]. *PCYT2* activity has been shown to be increased in breast cancer cells, enabling them to adapt to metabolic stress [45], and *TRERF1* acts as a cell cycle inhibitor in breast cancer cells through the modulation of progesterone receptor [46]. Altogether, this data supports the hypothesis of a key role for lipid metabolism deregulation in this case of ovarian HGSC. Interestingly, TCGA expression data regarding ovarian cancer shows that the expression of lipid metabolism-related genes is consistently altered in this type of tumor [47]. As a steroid hormone producing organ, steroid metabolism must be tightly regulated in the ovary. Besides, it is well known that lipogenesis is increased in cancerous cells in comparison to normal cells. In addition, lipid catabolism inhibits glycolisis, the major energy source used by malignant cells. Moreover, *TP53* has been shown to be a key regulator of lipid metabolism, inhibiting its anabolism and promoting its catabolism through the regulation of gene expression [48]. Thus, lipid metabolism could represent an important metabolic pathway related to ovarian cancer progression, constituting a new therapeutic target for this type of cancer, frequently resistant to conventional therapy.

Nevertheless it is important to take into consideration that this study has been carried out with a single patient. In order to strengthen its conclusions about the consequences of intra-tumoural heterogeneity in HGSCs, and also to gain a deeper understanding of the biology of p53-null HGSCs, an in-depth study considering a larger number of patients should be undertaken.

## Conclusions

Overall, these data shed light on the clonal evolution of the distinct tumor regions and thus on at least some of the genes that may be implicated in its progression and recurrence, and highlights the importance of considering intra-tumor heterogeneity when carrying out genetic and genomic studies, especially when these are aimed to diagnostic procedures or to uncover possible therapeutic strategies.

## Additional files

Additional file 1: **Supplementary Materials: Whole-exome sequencing: bioinformatics analysis.** (PDF 39 kb)

Additional file 2: Figure S1. Fluorescence in situ hybridisation of *PML* gene in primary tumor and recurrence samples shows genomic intra-tumoral heterogeneity. Representative FISH images of *PML* (red) and *RARA* (green, used as control) genes in primary tumor (P1-P6) and recurrence (IR1-IR2 and ER1-ER4) samples. Magnification = 40X. (PDF 115 kb)
