# Supplementary material for: Intra-tumor heterogeneity in TP53 null High Grade Serous Ovarian Carcinoma progression
Source: BMC Cancer. 2015 Nov 30;15:940. doi: 10.1186/s12885-015-1952-z (PMC4666042; doi:10.1186/s12885-015-1952-z)
Supplement: Supplementary file 1 — Supplementary Materials: Whole-exome sequencing: bioinformatics analysis. (PDF 39 kb) [file 12885_2015_1952_MOESM1_ESM.pdf]

Intra-tumor heterogeneity in *TP53* null High Grade Serous Ovarian Carcinoma progression

Alba Mota, Juan Carlos Triviño, Alejandro Rojo-Sebastián, Ángel Martínez-Ramírez, Luis Chiva, Antonio González-Martín, Juan F. Garcia, Pablo Garcia-Sanz, Gema Moreno-Bueno.

Supplementary Materials, Figures and Tables

Supplementary Materials

Whole-exome sequencing: bioinformatics analysis

Tumoral and normal samples were aligned against the human reference genome (version hg19) using paired-end library. Alignment was performed using Bioscope (http://solidsoftwaretools.com) and 'in house' scripts. The post-alignment sequencing filtering was performed using Picard-tools (http://picard.sourceforge.net) and SAMtools (http://samtools.sourceforge.net/). As standard quality metrics, percentage of mapped reads that passed quality filters (High Quality or HQ reads) and the percentage of reads 'on target' (reads aligned within target regions) were calculated. All samples presented more than 70% HQ reads and more than 60% reads 'on target', overtaking the minimum quality criteria for this type of sequencing. Coverage analysis revealed that more than 85% of exome positions had a minimum 10X coverage (**Supplementary Table 5**).

Supplementary Figure 1. Fluorescence *in situ* hybridisation of *PML* gene in primary tumor and recurrence samples shows genomic intra-tumoral heterogeneity. Representative FISH images of *PML* (red) and *RARA* (green, used as control) genes in primary tumor (P1-P6) and recurrence (IR1-IR2 and ER1-ER4) samples. Magnification = 40X

Supplementary Table1. Variants detected by whole-exome sequencing of HGSC. Sample distribution and variants consequence.

Sample	Gene	Variant	P1 Freq	IR1 Freq	ER1 Freq	Transcript Variant	PT Variant	Variant Conse-quence	Condel Prediction	SIFT Prediction	PolyPhen Prediction
Ubiquitous	ACADVL	chr17,7123949,G,A	0,625	0,551	0,798	c.300G>A	p.Met100Ile	Missense	neutral (0.012)	tolerated (0.46)	benign (0.002)
Ubiquitous	APOBEC 3H	chr22,39496336,G,T	0,316	0,118	0,231	c.53G>T	p.Arg18Leu	Missense	neutral (0.060)	tolerated (0.16)	benign (0.015)
Ubiquitous	BFSP2	chr3,133166157,C,A	0,536	0,348	0,421	c.490-4C>A	-	Splice	-	-	-
Ubiquitous	BIRC6	chr2,32743351,C,G	0,41	0,132	0,39	c.11380C>G	p.Gln3794Gl u	Missense	neutral (0.213)	tolerated (1)	possibly_damagin g (0.615)
Ubiquitous	CCDC129	chr7,31617618,A,G	0,348	0,239	0,403	c.818A>G	p.Lys273Arg	Missense	neutral (0.049)	tolerated (0.23)	benign (0.095)
Ubiquitous	CSMD3	chr8,113651011,G,T	0,561	0,395	0,5	c.3440C>A	p.Ala1147As p	Missense	deleterious (0.945)	deleterious (0)	probably_damagin g (1)
Ubiquitous	CTC1	chr17,8141496,G,T	0,696	0,706	0,667	c.500C>A	p.Pro167His	Missense	deleterious (0.859)	deleterious (0)	probably_damagin g (0.972)
Ubiquitous	DDX24	chr14,94526523,T,C	0,458	0,297	0,457	c.1834A>G	p.Ile612Val	Missense	neutral (0.019)	tolerated (0.37)	benign (0.014)
Ubiquitous	DSG2	chr18,29125970,C,G	0,361	0,393	0,435	c.2621C>G	p.Thr874Ser	Missense	neutral (0.021)	tolerated (0.36)	benign (0.011)
Ubiquitous	ETFDH	chr4,159624657,C,T	0,8	0,474	0,818	c.1199C>T	p.Thr400Ile	Missense	neutral (0.396)	tolerated (0.09)	benign (0.318)
Ubiquitous	FAM171 A1	chr10,15296860,C,T	0,5	0,367	0,562	c.437G>A	p.Arg146His	Missense	neutral (0.029)	tolerated (0.36)	benign (0.111)

Ubiquitous	FAP	chr2,163031418,C,A	0,12	0,266	0,386	c.1928G>T	p.Cys643Phe	Missense	deleterious (0.935)	deleterious (0)	probably_damaging (0.999)
Ubiquitous	FAT3	chr11,92523392,C,A	0,237	0,228	0,22	c.4611+8C>A	-	Splice	-	-	-
Ubiquitous	INSIG1	chr7,155093357,T,G	0,536	0,214	0,345	c.494T>G	p.Met165Arg	Missense	deleterious (0.565)	tolerated (0.06)	possibly_damaging (0.68)
Ubiquitous	KIAA1462	chr10,30336567,T,C	0,267	0,105	0,25	c.175A>G	p.Thr59Ala	Missense	neutral (0.001)	tolerated (0.79)	benign (0.002)
Ubiquitous	KIF21B	chr1,200972826,G,T	0,763	0,292	0,351	c.1100C>A	p.Ala367Asp	Missense	deleterious (0.945)	deleterious (0)	probably_damaging (1)
Ubiquitous	LAMA2	chr6,129465038,T,C	0,7	0,522	0,714	c.640-8T>C	-	Splice	-	-	-
Ubiquitous	MICAL3	chr22,18273682,T,A	0,36	0,278	0,357	c.5825A>T	p.Asp1942Val	Missense	deleterious (0.919)	deleterious (0)	probably_damaging (0.998)
Ubiquitous	MUC2	chr11,1093172,C,T	0,2	0,115	0,158	c.4991C>T	p.Thr1664Ile	Missense	-	-	unknown (0)
Ubiquitous	MYH15	chr3,108211438,C,G	0,406	0,404	0,724	c.840G>C	p.Leu280Phe	Missense	deleterious (0.487)	deleterious (0.02)	benign (0.333)
Ubiquitous	NOBOX	chr7,144095512,G,A	0,467	0,286	0,5	c.1637C>T	p.Pro546Leu	Missense	deleterious (0.850)	deleterious (0)	probably_damaging (0.963)
Ubiquitous	PABPC3	chr13,25670907,C,A	0,152	0,167	0,231	c.571C>A	p.Pro191Thr	Missense	neutral (0.000)	tolerated (1)	benign (0)
Ubiquitous	PABPC3	chr13,25670919,A,G	0,241	0,258	0,118	c.583A>G	p.Ile195Val	Missense	neutral (0.003)	tolerated (0.98)	benign (0.05)
Ubiquitous	PLEC	chr8,144991580,G,A	0,443	0,413	0,469	c.12820C>T	p.Arg4274Cys	Missense	-	deleterious (0)	unknown (0)
Ubiquitous	RINL	chr19,39367096,T,A	0,26	0,218	0,231	c.85A>T	p.Thr29Ser	Missense	neutral (0.029)	tolerated (0.29)	benign (0.005)
Ubiquitous	RNF17	chr13,25428199,C,T	0,389	0,219	0,344	c.3527C>T	p.Ala1176Va	Missense	neutral	tolerated	benign (0.003)

							l		(0.012)	(0.45)	
Ubiquitous	SLC5A12	chr11,26734256,A,G	0,414	0,276	0,484	c.340-3T>C	-	Splice	-	-	-
Ubiquitous	SMG7	chr1,183502437,T,A	0,868	0,219	0,347	c.982T>A	p.Trp328Arg	Missense	deleterious (0.839)	deleterious (0.04)	probably_damaging (0.999)
Ubiquitous	ST6GALNAC6	chr9,130653244,T,A	0,182	0,286	0,333	c.376A>T	p.Ile126Phe	Missense	deleterious (0.804)	deleterious (0)	probably_damaging (0.906)
Ubiquitous	SULT2B1	chr19,49096041,T,A	0,333	0,25	0,5	c.613T>A	p.Phe205Ile	Missense	neutral (0.405)	tolerated (0.35)	probably_damaging (0.939)
Ubiquitous	TAZ	chrX,153647890,G,T	0,211	0,25	0,417	c.469G>T	p.Val157Phe	Missense	deleterious (0.935)	deleterious (0)	probably_damaging (0.999)
Ubiquitous	*TP53*	chr17,7577579,G,C	1	0,588	0,667	c.702C>G	p.Tyr234Ter	Nonsense	-	-	-
Ubiquitous	TPO	chr2,1457512,C,A	0,449	0,258	0,41	c.529C>A	p.Leu177Ile	Missense	deleterious (0.895)	deleterious (0)	probably_damaging (0.993)
Ubiquitous	TRMT13	chr1,100614323,G,C	0,514	0,283	0,379	c.1393G>C	p.Val465Leu	Missense	deleterious (0.756)	deleterious (0.02)	probably_damaging (0.924)
Ubiquitous	TRO	chrX,54957346,G,A	0,233	0,154	0,157	c.4189G>A	p.Gly1397Ser	Missense	deleterious (0.906)	deleterious (0)	probably_damaging (0.996)
Ubiquitous	TTF2	chr1,117622279,T,A	0,297	0,469	0,405	c.1783+8T>A	-	Splice	-	-	-
Ubiquitous	UBR3	chr2,170782161,G,C	0,312	0,25	0,533	c.2055G>C	p.Trp685Cys	Missense	deleterious (0.855)	deleterious (0)	probably_damaging (0.968)
Ubiquitous	ZFAT	chr8,135613821,G,C	0,368	0,264	0,576	c.2141C>G	p.Ala714Gly	Missense	deleterious (0.699)	deleterious (0.01)	possibly_damaging (0.783)
Ubiquitous	ZNF519	chr18,14105235,T,A	0,31	0,292	0,5	c.1304A>T	p.Lys435Ile	Missense	deleterious (0.700)	deleterious (0.02)	possibly_damaging (0.832)
Ubiquitous	ZNF568	chr19,37441379,G,A	0,407	0,379	0,621	c.1324G>A	p.Ala442Thr	Missense	neutral (0.457)	deleterious (0)	benign (0.143)

Ubiquitous	ZNF813	chr19,53994586,A,C	0,104	0,103	0,212	c.1100A>C	p.Lys367Thr	Missense	neutral (0.039)	tolerated (0.34)	benign (0.174)
Ubiquitous	ZP4	chr1,238049169,C,T	0,776	0,324	0,271	c.857G>A	p.Ser286Asn	Missense	deleterious (0.595)	tolerated (0.14)	possibly_damaging (0.883)
Shared P1+IR1	BTK	chrX,100629564,A,T	0,226	0,111	0,083	c.200T>A	p.Val67Glu	Missense	deleterious (0.919)	deleterious (0)	probably_damaging (0.998)
Shared P1+IR1	DNAH5	chr5,13868103,TA,T	0,238	0,19	0,083	c.3835-3delT	-	Splice	-	-	-
Shared P1+IR1	FRMPD1	chr9,37745930,G,T	0,361	0	0,316	c.3901G>T	p.Glu1301Ter	Nonsense	-	-	-
Shared P1+IR1	METTL2A	chr17,60526635,A,G	0,28	0,211	0,074	-	-	3'UTR	-	-	-
Shared P1+IR1	MTHFD2P7	chr3,179184090,C,A	0,374	0,221	0	-	-	3'UTR	-	-	-
Shared P1+IR1	MUC21	chr6,30954301,G,A	0,22	0,133	0,043	c.349G>A	p.Ala117Thr	Missense	-	tolerated (1)	unknown (0)
Shared P1+IR1	NUDT2	chr9,34343413,A,T	0,414	0,154	0	c.419A>T	p.Gln140Leu	Missense	neutral (0.301)	tolerated (0.08)	benign (0.026)
Shared P1+ER1	CIT	chr12,120260783,C,T	0,1	0,03	0,2	c.958-6G>A	-	Splice	-	-	-
Shared P1+ER1	GUCY2F	chrX,108695251,C,G	0,368	0,04	0,216	c.1472+1G>C	-	Splice	-	-	-
Shared P1+ER1	MSLNL	chr16,830475,G,A	0,26	0,086	0,117	c.526C>T	p.Pro176Ser	Missense	-	-	benign (0.006)
Shared P1+ER1	PCYT2	chr17,79862749,T,G	0,125	0,062	0,222	c.1184A>C	p.Gln395Pro	Missense	deleterious (0.561)	tolerated (0.05)	possibly_damaging (0.619)
Shared	PNMAL1	chr19,46973071,G,A	0,111	0	0,208	c.1222C>T	p.Gln408Ter	Nonsense	-	-	-

P1+ER1											
Shared P1+ER1	SETX	chr9,135152540,C,G	0,192	0	0,242	c.6843-1G>C	-	Splice	-	-	-
Shared P1+ER1	VEPH1	chr3,156983349,G,C	0,192	0,095	0,379	c.2231C>G	p.Ala744Gly	Missense	deleterious (0.818)	deleterious (0.02)	probably_damaging (0.986)
Shared IR1+ER1	FAM47C	chrX,37028662,T,C	0,054	0,107	0,263	c.2179T>C	p.Cys727Arg	Missense	-	tolerated (0.9)	unknown (0)
Shared IR1+ER1	HEPHL1	chr11,93834432,TCCATCTCAGC,T	0	0,164	0,2	c.2507_2516delCCATCTCAGC	p.Ile837ArgfsTer16	Frameshift	-	-	-
Shared IR1+ER1	KIF21A	chr12,39688273,G,A	0	0,243	0,333	c.4978C>T	p.Gln1660Ter	Nonsense	-	-	-
Shared IR1+ER1	MYH3	chr17,10533598,C,T	0,056	0,167	0,2	c.5457+7G>A	-	Splice	-	-	-
Shared IR1+ER1	OR56B1	chr11,5757874,T,C	0	0,325	0,357	c.128T>C	p.Leu43Pro	Missense	deleterious (0.867)	deleterious (0)	probably_damaging (0.979)
Shared IR1+ER1	SLC15A2	chr3,121641882,C,T	0	0,263	0,434	c.868-5C>T	-	Splice	-	-	-
Shared IR1+ER1	SLC39A11	chr17,70845949,CAAA,C	0,062	0,2	0,278	c.452-9_452-7delTTT	-	Splice	-	-	-
Shared IR1+ER1	ZNF664	chr12,124497296,G,A	0,062	0,12	0,261	c.605G>A	p.Cys202Tyr	Missense	neutral (0.383)	deleterious (0.02)	benign (0.008)
P1 Specific	CNOT1	chr16,58589390,C,T	0,317	0	0	c.2656G>A	p.Glu886Lys	Missense	deleterious (0.526)	deleterious (0.04)	possibly_damaging (0.526)
P1 Specific	IPO8	chr12,30805228,A,G	0,212	0,048	0,049	c.2075-5T>C	-	Splice	-	-	-
P1 Specific	MYBPC2	chr19,50946763,G,C	0,212	0	0	c.915G>C	p.Glu305Asp	Missense	deleterious (0.867)	deleterious (0.01)	probably_damaging (0.996)

P1 Specific	NPAP1	chr15,24922726,T,C	0,671	0	0	c.1712T>C	p.Val571Ala	Missense	-	-	benign (0.158)
P1 Specific	NR2C1	chr12,95425118,T,C	0,222	0,042	0,043	c.1393+7A>G	-	Splice	-	-	-
P1 Specific	PCDHA7	chr5,140215999,G,T	0,452	0	0	c.2031G>T	p.Lys677Asn	Missense	neutral (0.365)	deleterious (0.03)	benign (0.016)
P1 Specific	PLXNA1	chr3,126736607,C,A	0,309	0	0,013	c.3532C>A	p.Pro1178Thr	Missense	deleterious (0.857)	deleterious (0.02)	probably_damaging (0.998)
P1 Specific	RBMXL3	chrX,114426846,C,T	0,25	0,05	0,069	c.2842C>T	p.Arg948Cys	Missense	deleterious (0.621)	deleterious (0)	possibly_damaging (0.523)
P1 Specific	TRAPPC10	chr21,45523230,G,T	0,205	0	0	c.3598G>T	p.Asp1200Tyr	Missense	deleterious (0.884)	deleterious (0)	probably_damaging (0.989)
P1 Specific	TRERF1	chr6,42196294,G,A	0,39	0	0	c.3392C>T	p.Thr1131Met	Missense	-	-	probably_damaging (0.966)
P1 Specific	WIF1	chr12,65462673,C,A	0,219	0,062	0,038	c.409G>T	p.Gly137Cys	Missense	deleterious (0.556)	tolerated (0.18)	probably_damaging (1)
P1 Specific	ZNF71	chr19,57132631,T,C	0,2	0,056	0	c.-20-5T>C	-	Splice	-	-	-
P1 Specific	ZNF792	chr19,35449350,C,A	0,213	0,089	0,062	c.1409G>T	p.Arg470Leu	Missense	deleterious (0.806)	deleterious (0.04)	probably_damaging (0.995)
P1 Specific	BZRAP1	chr17,56388217,G,A	0,278	0,045	0,038	c.3439C>T	p.Pro1147Ser	Missense	neutral (0.031)	tolerated (0.29)	benign (0.035)
P1 Specific	DPCR1	chr6,30917617,G,A	0,25	0,095	0,042	c.1376G>A	p.Gly459Glu	Missense	neutral (0.000)	tolerated (1)	benign (0.002)
P1 Specific	DPP6	chr7,154561192,G,A	0,234	0	0	c.949G>A	p.Ala317Thr	Missense	neutral (0.000)	tolerated (1)	benign (0.001)
P1 Specific	KPNB1	chr17,45734400,G,A	0,489	0	0	c.457G>A	p.Ala153Thr	Missense	neutral (0.018)	tolerated (0.4)	benign (0.032)
P1 Specific	SIRPA	chr20,1895835,C,T	0,222	0	0,087	c.170C>T	p.Ala57Val	Missense	neutral	tolerated (1)	benign (0.002)

									(0.000)			
P1 Specific	UBE2D4	chr7,43978051,G,A	0,37	0	0	c.46G>A	p.Asp16Asn	Missense	neutral (0.312)	tolerated (0.1)	benign (0.253)
P1 Specific	YTHDC1	chr4,69199104,T,C	0,2	0	0,048	c.895A>G	p.Lys299Glu	Missense	neutral (0.015)	tolerated (0.41)	benign (0.008)
P1 Specific	ZSWIM2	chr2,187703865,C,G	0,242	0	0	c.315G>C	p.Glu105Asp	Missense	neutral (0.305)	tolerated (0.08)	benign (0.078)
IR1 Specific	CRYBG3	chr3,97605469,T,C	0,062	0,263	0,048	c.1309-6T>C	-	Splice	-	-	-
IR1 Specific	FAM73A	chr1,78245411,T,G	0	0,2	0	c.71T>G	p.Leu24Arg	Missense	neutral (0.445)	deleterious (0)	benign (0)
IR1 Specific	GAB3	chrX,153908522,C,T	0	0,289	0,023	c.1534G>A	p.Glu512Lys	Missense	neutral (0.355)	deleterious (0.04)	benign (0.086)
IR1 Specific	GTF2I	chr7,74119488,G,A	0	0,235	0	c.587-8G>A	-	Splice	-	-	-
IR1 Specific	KMT2A	chr11,118342635,A,G	0,08	0,2	0	c.761A>G	p.Lys254Arg	Missense	neutral (0.447)	deleterious (0)	benign (0.037)
IR1 Specific	PLEKHH 1	chr14,68050751,G,T	0,033	0,25	0,033	c.3609G>T	p.Leu1203Ph e	Missense	deleterious (0.849)	deleterious (0.04)	probably_damagin g (1)
IR1 Specific	ROBO2	chr3, 77147356, C, A	0,006	0,139	0	c.301C>A	p.Leu101Met	Missense	deleterious (0.880)	deleterious (0.01)	probably_damagin g (0.998)
IR1 Specific	TFDP1	chr13,114290294,A,C	0	0,2	0	c.690A>C	p.Gln230His	Missense	deleterious (0.534)	tolerated (0.21)	probably_damagin g (0.999)
IR1 Specific	TMEM97	chr17,26653815,A,T	0,071	0,2	0,033	c.527A>T	p.Lys176Ile	Missense	-	deleterious (0)	unknown (0)
IR1 Specific	ZAN	chr7,100373391,T,G	0	0,212	0	c.6125T>G	p.Val2042Gl y	Missense	deleterious (0.629)	deleterious (0)	possibly_damagin g (0.545)
ER1 Specific	BARX1	chr9,96715084,G,A	0	0	0,404	c.527C>T	p.Ala176Val	Missense	deleterious	deleterious	probably_damagin

									(0.945)	(0)	g (1)
ER1 Specific	CDC25A	chr3,48207388,A,T	0	0	0,2	c.1030-5T>A	-	Splice	-	-	-
ER1 Specific	ECE1	chr1,21584014,T,G	0,02	0,091	0,263	c.828+4A>C	-	Splice	-	-	-
ER1 Specific	GPNMB	chr7,23313805,C,T	0	0	0,688	c.1681C>T	p.Pro561Ser	Missense	deleterious (0.743)	tolerated (0.13)	probably_damagin g (0.999)
ER1 Specific	HYKK	chr15,78825713,A,T	0,026	0	0,2	c.823A>T	p.Ile275Phe	Missense	deleterious (0.532)	deleterious (0.04)	possibly_damagin g (0.542)
ER1 Specific	PLEKHS1	chr10,115537025,C,T	0	0	0,25	c.1167+7C>T	-	Splice	-	-	-
ER1 Specific	TRAK2	chr2,202251064,C,T	0	0	0,308	c.1840G>A	p.Glu614Lys	Missense	neutral (0.390)	deleterious (0.02)	benign (0.086)

Supplementary Table 2. PCR conditions for Sanger analysis of variants detected by whole-exome sequencing

Gene	Forward primer (5' to 3')	Reverse primer (5' to 3')	Length	Tm	Mutation validation
**CNOT1**	TGCGTTGTTTTTGTCTTGCT	AGCCAGACCTAGTGCCATGT	225	58	YES
**CSMD3**	TGAATGAGCCCTTTTGTTTTT	GGCAGTTTTACCCAACCACT	180	58	YES
**CTC1**	TCAAAGGAAACACTGGCACA	CCCTTGCTCTTGGTCTTTCTT	162	58	YES
**ECE1**	ACACGAATTCCCTCTCATGC	CCTGTTTCACCCATCAGGTC	234	58	NO
**FAP**	TTCTCCAGCTCCCTTCAGTC	CGTGTTAAATGCTTTCACAGTAACA	250	58	YES
**FRMPD1**	CAGAAGGCAAAAGTGACAGC	CCTGCAAAACCCAAAGATGT	127	58	YES
**GPNMB**	TCAGAAGCAAAGGCCTGAGT	CAACTTCCCCAAACCACAAT	235	58	YES
**GTF2I**	GTGTGATCCAGAGCTGCAAA	AGGTGTGGGAGTTAAACAGCA	214	58	NO
**HEPHL1**	GGCCTGTGTTTTGCCTTTAG	CTGGTTTTGTCATGGGCACT	161	58	YES
**KIF21A**	ATCACAGCATTCAGTTTACAACC	TCTGCCCTTGTTCATATATCCAT	176	58	YES
**KIF21B**	TCACAACAGCCTTTCTGCAC	ACTGATTTGCTGGCTGGTCT	168	58	YES
**KMT2A**	GAGGAAGACCTCCCACCTTC	TTTGTACCCCCTTCCTTCCT	231	58	NO
**LAMA2**	CCAGTGCATAGGCATGTACC	GCACTTGGTCTCCCATTGAT	176	58	YES
**OR56B1**	CATTCACAGCTGGCAACACT	GCCAGACCCATGTCTACCAT	165	58	YES
**PCYT2**	CAGAAGAAGGAAGCCAAGGA	CAAGGAGGCAGAGTCCTCAC	223	58	NO
**PLXNA1**	CGTACTGGAGCCACTCAGC	CGATGAGCACCGTGTAGTTG	212	58	YES
**ROBO2**	TACAAAGATGGGGAGCGAGT	CCAAGATAGTTCCTCGCAACA	209	58	YES
**SMG7**	TTTAGCAATGAAACCGAGCA	GCTTTGCTACATCGATGAAATG	153	58	YES
**TFDP1**	AGATGTCCAGGCCAACTCCT	CTTCTTGCTGGTGTTGACGA	241	58	YES
**TP53**	CCACAGGTCTCCCCAAGG	TGGCAAGTGGCTCCTGAC	183	55	YES
**TRERF1**	TCCACATCCTTGATGGGTTT	ACAGAGGCAAAAGGCTCAGA	138	58	YES
**UBE2D4**	CACCAGGAGAATTCCTTCCA	ACTTACAGTCATCACCGACAGG	162	58	YES
**UBR3**	TTGGACAGAATATCGGGCTTA	TGGACATACGTCATGGCTTG	202	58	YES
**ZFAT**	CAGCAGGTGTCTCAGGTCAA	GCTGCCTTTCCTTACCACAG	248	58	YES
**ZNF664**	ACACCTCCAGCCTCTGCAT	AGGCCTTTCCGCACTCAT	246	58	YES

Supplementary Table 3. Copy Number Variants detected by Comparative Genomic Hybridisation

Enhanced		Diminished	
**Region**	**Sample**	**Region**	**Sample**
1p22p35	P1+ER1	-4	ALL
2q32q33	ALL	6q25q27	ALL
3q22q29	ALL	8p22p23	P1
5p15	ER1	-12	P1+ER1
7q22q32	ALL	-16	P1+ER1
8q12q24	ALL	16q24	IR1
9q31	P1+ER1	17p13	P1+ER1
10q22	ER1		
11q14q22	ALL		
15q22q26	P1		
22q13	ER1		

Supplementary Table 4. Functional annotation of selected signaling pathways

Category	Term	PValue	Genes
BIOCARTA	Cyclins and Cell Cycle Regulation	0.008	CDK7, CDC25A, TFDP1
BIOCARTA	Cell Cycle: G1/S Check Point	0.010	TP53, CDC25A, TFDP1
COG ONTOLOGY	Cell division and chromosome partitioning	0.194	MYH3, CIT, PLEC
GOTERM_BP_ALL	GO:0007155: Cell adhesion	0.003	LAMA2, PCDHA7, MYBPC2, DSG2, FAT3, TRO, ZAN, ROBO2, MSLNL, GPNMB, SIRPA
GOTERM_BP_ALL	GO:0022610: Biological adhesion	0.003	LAMA2, PCDHA7, MYBPC2, DSG2, FAT3, TRO, ZAN, ROBO2, MSLNL, GPNMB, SIRPA
GOTERM_BP_ALL	GO:0007156: Homophilic cell adhesion	0.004	PCDHA7, DSG2, FAT3, TRO, ROBO2
GOTERM_BP_ALL	GO:0016337: Cell-cell adhesion	0.013	PCDHA7, DSG2, FAT3, TRO, ZAN, ROBO2
GOTERM_BP_ALL	GO:0000059: Protein import into nucleus, docking	0.083	IPO8, KPNB1
GOTERM_BP_ALL	GO:0007018: Microtubule-based movement	0.113	KIF21A, KIF21B, DNAH5
GOTERM_BP_ALL	GO:0006629: Lipid metabolic process	0.120	ACADVL, ST6GALNAC6, TAZ, INSIG1, ETFDH, SULT2B1, PCYT2, TRERF1
GOTERM_BP_ALL	GO:0006635: Fatty acid beta-oxidation	0.134	ACADVL, ETFDH
GOTERM_BP_ALL	GO:0009062: Fatty acid catabolic process	0.168	ACADVL, ETFDH
GOTERM_BP_ALL	GO:0019395: Fatty acid oxidation	0.181	ACADVL, ETFDH
GOTERM_BP_ALL	GO:0034440: Lipid oxidation	0.181	ACADVL, ETFDH
GOTERM_BP_ALL	GO:0008283: Cell proliferation	0.182	INSIG1, TP53, CDK7, CDC25A, TFDP1
GOTERM_BP_ALL	GO:0006351: Transcription, DNA-dependent	0.187	GTF2I, TP53, CDK7, TTF2
GOTERM_BP_ALL	GO:0006355: Regulation of transcription, DNA-dependent	0.187	ZNF519, ZNF568, TP53, ZNF813, ZNF792, CDK7, TRERF1, NR2C1, BARX1, TRAK2, ZNF71, NOBOX, TFDP1
GOTERM_MF_FAT	GO:0003774: Motor activity	0.008	MYH15, MYH3, KIF21A, KIF21B, DNAH5
GOTERM_MF_FAT	GO:0003777: Microtubule motor activity	0.068	KIF21A, KIF21B, DNAH5
GOTERM_MF_FAT	GO:0004386: Helicase activity	0.182	DDX24, TTF2, SETX
KEGG PATHWAY	hsa04110: Cell cycle	0.020	TP53, CDK7, CDC25A, TFDP1
REACTOME PATHWAY	REACT_578: Apoptosis	0.109	DSG2, KPNB1, PLEC

Supplementary Table 5. Quality metrics of whole-exome sequencing data

Sample	Number of Reads	% 'HQ' Reads	% Reads ‘on target’	% Exome positions with coverage >10
N	73640690	70,84	60,14	89,12
P1	73200254	70,44	60,42	90,4
IR1	62298712	71,59	61,68	91,65
ER1	71276218	70,98	61,22	93,31
