# Supplementary figures and images for: Intra-tumor heterogeneity in TP53 null High Grade Serous Ovarian Carcinoma progression
Source: BMC Cancer. 2015 Nov 30;15:940. doi: 10.1186/s12885-015-1952-z (PMC4666042; doi:10.1186/s12885-015-1952-z)

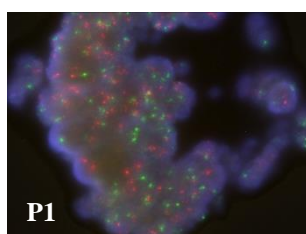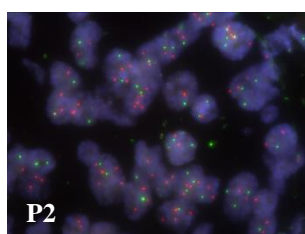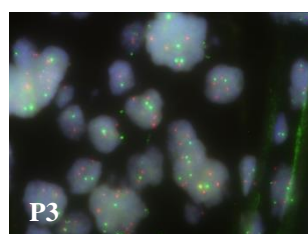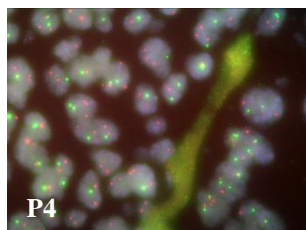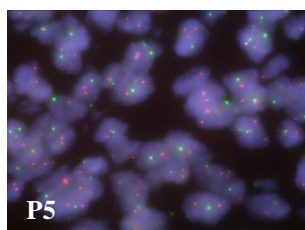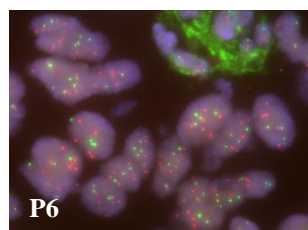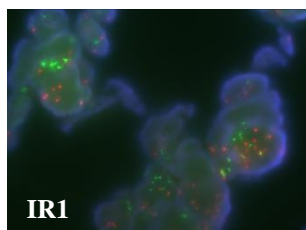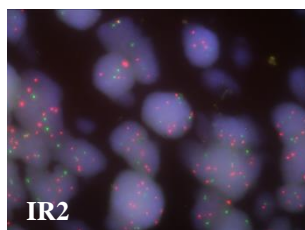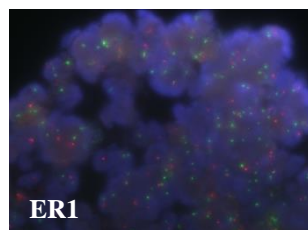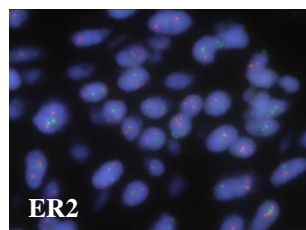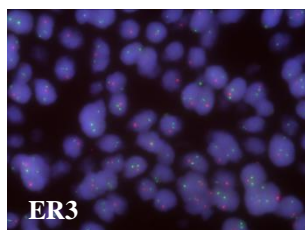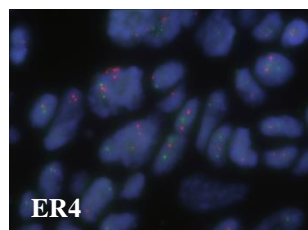

Supplement: Supplementary file 2 — Fluorescence in situ hybridisation of PML gene in primary tumor and recurrence samples shows genomic intra-tumoral heterogeneity. Representative FISH images of PML (red) and RARA (green, used as control) genes in primary tumor (P1-P6) and recurrence (IR1-IR2 and ER1-ER4) samples. Magnification = 40X. (PDF 115 kb) [file 12885_2015_1952_MOESM2_ESM.pdf]
